# Taxonomic Review of the Orders Mysida and Stygiomysida (Crustacea, Peracarida)

**DOI:** 10.1371/journal.pone.0124656

**Published:** 2015-04-30

**Authors:** Kenneth Meland, Jan Mees, Megan Porter, Karl J. Wittmann

**Affiliations:** 1 Department of Biology, University of Bergen, Bergen, Norway; 2 Flanders Marine Institute and Ghent University, Ostend, Belgium; 3 Department of Biology, University of South Dakota, Vermillion, South Dakota, United States of America; 4 Medizinische Universität Wien, Institut für Umwelthygiene, Abteilung Ökotoxikologie, Vienna, Austria; Ecole normale superieure de Lyon, FRANCE

## Abstract

The order Mysida (2 families, 178 genera, 1132 species) contains species across a broad range of habitats, such as subterranean, fresh, brackish, coastal, and surface to deep-sea habitats. The Stygiomysida (2 families, 2 genera, 16 species), however, are found primarily in subterranean waters, but always in waters with a marine influence. The Mysida and Stygiomysida body is divided into three main regions: cephalon, thorax, and abdomen. They are shrimp-like in appearance, containing morphological features earlier referred to as defining a "*caridoid facies*". The shrimp-like morphology was to some extent diagnostic for the historic Decapod taxon Schizopoda, containing the Nebalia, Mysida, Lophogastrida, and Euphausiacea. In 1904 the concept of Schizopoda was abandoned, and the Mysidacea (Mysida and Lophogastrida) along with Cumacea, Amphipoda, Isopoda, and Tanaidacea were placed in a new taxon, the Peracarida. Later discoveries of groundwater mysids led to the establishment of Stygiomysida, but placement to either Lophogastrida or Mysida remained unclear. The presence of oostegites and absence of podobranchiae, coupled with non-statocyst bearing uropods have been used to classify the Stygiomysida as a primitive Mysida family, comparable to Petalophthalmidae. On the other hand, equally suggestive characters, but for a Lophogastrida affiliation, was suggested for the archaic foregut characters and again, non-statocyst bearing uropods. With the inclusion of DNA sequence data of ribosomal genes, sister group relationships between Stygiomysida, Lophogastrida, and Mictacea within the Peracarida are observed, which supports a classification of the Stygiomysida as a separate order removed from the Mysida.

## Introduction

The orders Mysida and Stygiomysida, together with the Lophogastrida, earlier referred to as the “Mysidacea” consists of approximately 1200 described species and 187 genera found across all latitudes throughout the waters of the world, with the majority of species inhabiting coastal and open ocean waters. Extrapolations of the global biodiversity within the Mysidacea, however, propose upwards of 4000 species, suggesting that there are many more species yet to be discovered [1]. Species within this group are generally pelagic or epi- to hyperbenthic, omnivorous filter feeders, ranging in size from 5–25 mm. The Mysidacea demonstrate a set of shrimp-like characters known as the “*caridoid facies*” [2]. Distinguishing features include the presence of a statocyst in the uropod and the presence of a marsupium (brood pouch) in females. Although the first mysidacean species were described in the 18^th^ century [3], over two hundred years later there is still debate over the taxonomic organization of the group, as well as where the Mysidacea fit within the Crustacea. Although currently classified within the Peracarida, at various times the Mysidacea have been allied with the euphausiids, the decapods, the stomatopods, and even the nebaliaceans (see Tattersall & Tattersall [4] for taxonomic history). More recently, a number of studies have begun to investigate the phylogenetic relationships among the Mysidacea. This recent phylogenetic research has shown that the ‘Mysidacea’ consists of several distinct orders—the Mysida, the Lophogastrida, and a new order, the Stygiomysida [5, 6]. The order Mysida (2 families, 178 genera, 1132 species) contains the largest number of species across the greatest diversity of habitats, with species found in subterranean, fresh, brackish, coastal, and surface to deep-sea habitats. In comparison, the Lophogastrida (3 families, 7 genera, 54 species) are mainly meso- to bathypelagic while the Stygiomysida (2 families, 2 genera, 16 species) are found primarily in subterranean waters.

In the following review we focus on presenting comparative morphology between, and the taxonomic history of, the taxa, Mysida and Stygiomysida. The taxonomic rank of order in the Stygiomysida has been proposed by Meland & Willassen [6] and implemented in the World Register of Marine Species [7]. Nonetheless, besides molecular phylogenetic based hypothesis, the Stygiomysida have until now not received a proper taxonomic revision to justify a re-classification as an order removed from the Mysida, as listed in the “World List of Lophogastrida, Stygiomysida and Mysida” (see below).

The World List of Lophogastrida, Stygiomysida and Mysida [5] is part of the WoRMS, a global initiative to provide a register of all marine organism names. This world list aims to (1) provide an authoritative catalogue of the world's lophogastrid, stygiomysid and mysid species, (2) promote stability in nomenclature, (3) act as a tool for higher taxonomic revisions and regional monographs, (4) provide a base link for other online databases, and (5) provide additional information—e.g. distribution records—for all species. Although the treatment in three separate orders is now accepted, all mysid, lophogastrid and stygiomysid species ever described are presented in the same web interface. The higher classification (orders, families, subfamilies) follows Meland & Willassen [6], with additions by Wittmann et al. [8]. Fossil records, including the entirely fossil order of Pygocephalomorpha, are also included in the database, and the same holds for freshwater, commensal, groundwater and cave species.

## External Morphology

**Fig 1:**
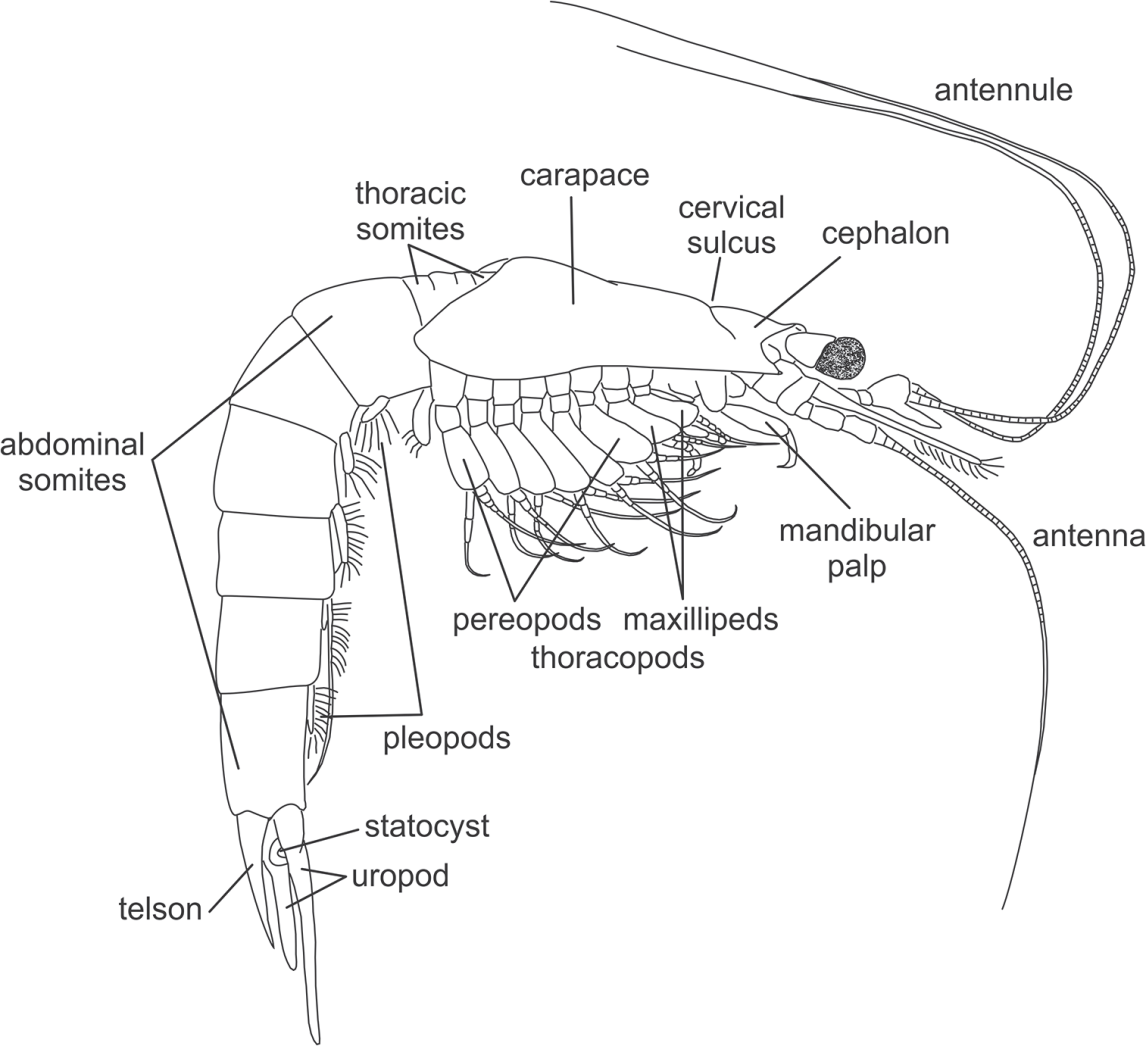
External morphology of a typical Mysida male.

The Mysida and Stygiomysida body is divided into three main regions: cephalon (5 somites, or a total of 6 somites including a putative ocular segment [9, 10]), thorax (8 somites), and abdomen (6 somites) (Fig 1). They are shrimp-like in appearance, containing features earlier referred to as defining a "*caridoid facies*" shared by the Euphausiacea, Lophogastrida, and Decapoda [2, 11]. The facies features comprise: 1) carapace enveloping the thorax, 2) movable stalked eyes, 3) biramous antennules, 4) scale-like antennal exopods, 5) natatory exopods on the thoracopods, 6) elongate, ventrally flexible abdomen, 7) tail-fan formed by uropods and telson, 8) trunk musculature serving strong ventral flexion, 9) internal organs mainly excluded from abdomen, 10) pleopods 1–5 biramous. Most of the characters attributed to the "*caridoid facies*" are considered plesiomorphic states within the Malacostraca, but within the mysidacean orders variation in "*caridoid facies*" characters are often quite useful as diagnostics for defining the higher taxa from families to genera, and in many cases also genera and species. The following character descriptions highlight morphological variations useful for taxon specific diagnostics within Stygiomysida and Mysida.

### Carapace

The Mysida carapace is fused with no more than the first four anterior somites of the thorax and is posteriorly produced to form lateral flaps; dorsally the carapace emarginates leaving the last thoracic somites exposed. Fusion with the thoracic somites is indicated by a “cervical sulcus” running across the carapace in the vicinity of the mandibles. The inner lateral walls of the carapace are membranous and are respirarory in function. In the family Stygiomysidae the carapace does not expand beyond the 5th somite, encompassing only the cephalic region. In the Lepidomysidae (Stygiomysida) it extends to the 7th somite or beyond.

The anterior margin of the carapace may be evenly arcuate or forwardly extended forming a well-defined rostrum. There is considerable variation in rostrum shape and size but it is commonly not seen to extend much further than the ocular papilla.

### Cephalon

**Fig 2:**
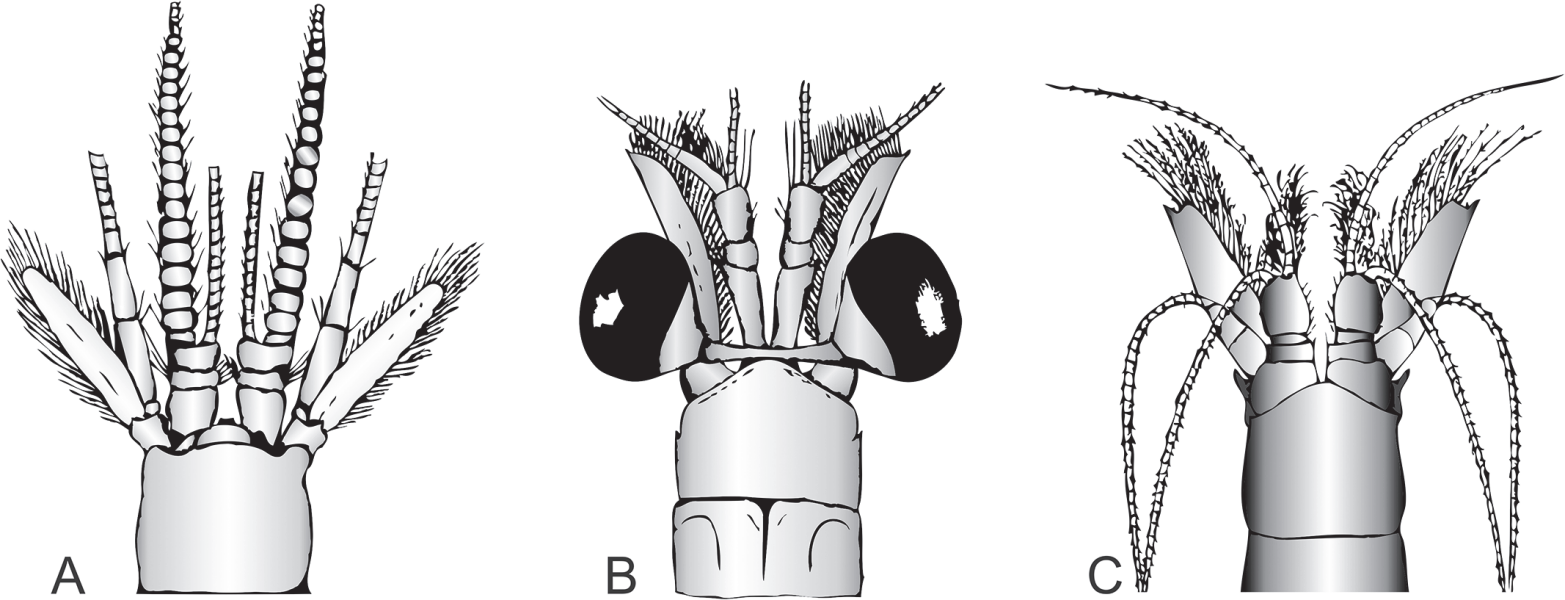
Mysida cephalon. (A) *Hansenomysis fyllae* (Hansen, 1887). (B) *Boreomysis megalops* G.O. Sars, 1872, (C) *Amblyops kempi* (Holt & Tattersall, 1905).

**Eyes** In the majority of mysid species the eyes are stalked and movable, displaying a wide range of shapes and sizes (Fig 2). In many species the eyestalk bears a dorsally placed ocular papilla, and this finger-like process is often very long and well developed in deep-sea and pelagic species. The cornea's pigmentation varies from black, golden, red-brown, to brilliant red. Complete reduction of the cornea, with some genera showing no traceable visual elements, is often seen in deep water forms (e.g. species of the subfamily Erythropinae). Here the eyes can take on the form of two flattened plates (*Amblyops*), and these sometimes fuse to form a single eyeplate (*Pseudomma*). Reduction in eye morphology is also seen in the cavernicolous Stygiomysida and the deep water Petalophthalmidae. Here, separate, well-developed or vestigial eyestalks are present, but the cornea is reduced to a few ommatidia or is completely missing.

**Antennule** The antennule sympod, also termed the peduncle, consists of three segments: precoxa, coxa, and basis. In Mysida the sympod supports normally two flagella, with the outer flagellum usually longer than the inner one (a third flagellum-like process is found in species of *Mesopodopsis*). Sexual dimorphism in the flagella is common, being larger and more robust in males compared to females. This is most conspicuous in the Petalophthalmidae genus *Hansenomysis* (Fig 2A). In the Mysidae, sexual dimorphism is also seen in mature males having an anteriorly produced ventral process on the distal end of the third segment of the sympod. This lobe is often referred to as the *appendix masculina* and is densely covered with long sensory setae (Fig 2C)

**Antenna** The sympod of the antenna is closely fused, and the delimitations between the praecoxa, coxa, and basis, are not always easily made out. The exopod normally takes on the form of an antennal scale (antennal plate), with margins entirely or partially set with long plumose setae (Fig 2A). In some cases the outer margin is naked and terminates in a distal articulated or non-articulated spine (Fig 2B). In certain genera of the Erythropinae the outer margins are serrated, devoid of setae, while in *Hansenomysis* (family Petalophthalmidae) marginal spines are dispersed between the outer setae (Fig 2A). In most mysids the antennal scale is divided distally by either a transverse or oblique suture, missing in some species of Erythropinae, Leptomysinae, and Mysinae. The antennal scale is reduced to a very small plate or spine in the Stygiomysidae and Palaumysinae, and also in some genera within the Erythropinae. When combining these characters, antennal scale morphology can be very informative in distinguishing higher Mysida taxa. The antennal endopod takes on the form of a multi-segmented flagellum. Its first three proximal segments are termed the antennal peduncle and are always much larger than the remaining distal segments (Fig 2C).

**Labrum** The mouth field is anteriorly closed by the labrum that represents a hump-like plate, mostly broader than long. However, in the Siriellinae, Gastrosaccinae, and in certain Mysinae it extends into a long anteriorly directed process. The labrum shows often asymmetrical sets of setae and spines but its general form is normally about symmetrical—except for the Mysidellinae where it extends posteriorly into two strongly asymmetric processes.

**Fig 3:**
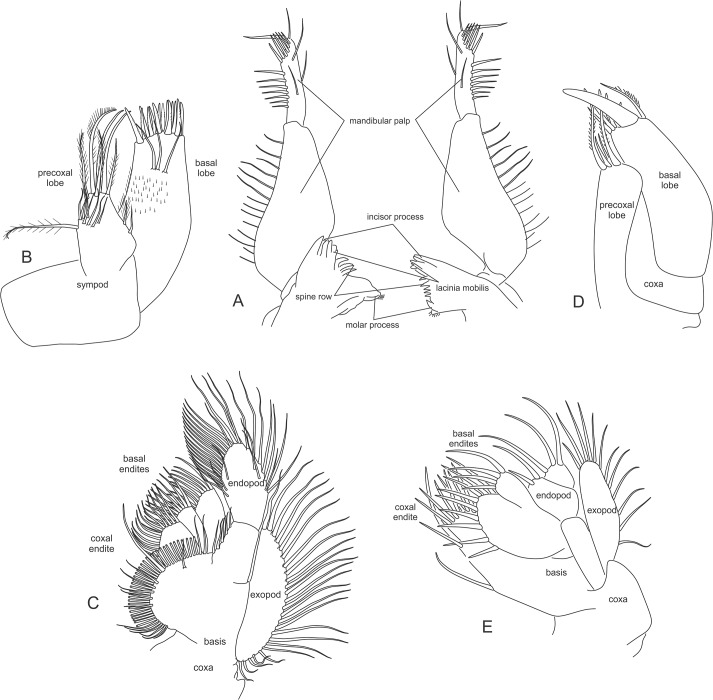
Mouthpart morphology of Mysida. (A) left and right mandibles. (B) maxillule, *Pseudomma antarcticum* Zimmer, 1914. (C) maxilla, *Pseudomma antarcticum*. (D) maxillule, *Stygiomysis holthuisi* (Gordon, 1958). (E) maxilla, *Stygiomysis holthuisi*.

**Mandible** The Mysida mandible is well developed, usually large and heavily chitinized. Note that the gnathobasic processes of the left and right mandibles are not alike, but both mandibles take on a typical peracarid form consisting of four structures (Fig 3A).

**Incisor process** Placed distally and consisting of a series of cusps or teeth forming a serrated sharp ridge. The incisor is always well developed in all the Mysida.

**Lacinia mobilis** Inserted proximal to the incisor process, the lacinia mobilis displays noticeable differences in the right and left mandible. In the Petalophthalmidae the lacinia mobilis is missing, probably reduced. A similar reduction is also seen in the deep sea species *Mysimenzies hadalis*.

**Spine row (= pars centralis)** The space between the lacinia mobilis and molar process is more or less taken up by a row of spines. These spines can give this area a jagged, comb-like appearance or display only a few serrated spines with complex denticles, very often differing in the left and right mandibles. Associated with the reduced lacinia mobilis one can observe a complete reduction of the spine row in the Lophogastrida families and a reduction to a single spine in the Petalophthalmidae. Although still supporting a well-developed lacinia mobilis, absence of a spine row is also observed in *Gastrosaccus*.

**Molar process** With the exception of the reduced molar process seen in Siriellinae and in certain Leptomysinae (*Mysidopsis*), the typical mysid molar process is observed as a flat plate-like structure provided with fine ridges or spines.

During development the mandible exopods seen in the nauplioid (= first larval stage in the marsupium) are lost, while the naupliar sympod and endopod take on the form of a three-segmented palp. The first segment is very small and is often overlooked. The second and third segments are long and armed with robust setae, the palp functions as a tool to transfer food into the mouth and scrape food from the surrounding mouthparts. In the raptorial Petalophthalmidae genus *Petalophthalmus* the palp is highly modified, taking on the form of a powerful prehensile tool used to capture their prey.

**Labium** The mouth field is posteriorly closed by the labium that represents densely setose, bilaterally roughly symmetric paragnaths with a more or less distinct common base. The setae may be in part quite stiff, spine-like. The Erythropinae genus *Thalassomysis* is exceptional among the Mysida by its asymmetric paragnaths with a large common base. In the Stygiomysida genus *Stygiomysis* the paragnaths are long and apically widely separated. The Lophogastrida have generally more asymmetric paragnaths clearly showing masticatory structures.

**Maxillule** In the Mysida the morphology of the maxillule is quite uniform in form and function (Fig 3B), made up of a sympod, consisting of the praecoxa, coxa, and basis with appurtenant lobes. The praecoxa's lobe has a broad base and narrows to an obtusely pointed apex, furnished with long plumose setae. The praecoxa also supports a small leaf-like, mostly setose epipod (not visible in Fig 3), termed “pseudexopodite” by Nouvel et al. [12]. The coxa is very small and bears no lobe or setae. The basis is drawn out into a long inwardly directed lobe, bearing no marginal setae, and ending in a truncate apex, usually armed with two rows of strong spinose setae or teeth. In the Stygiomysida family Lepidomysidae, the sympod supports a two-segmented endopod/palp (Fig 3D).

**Maxilla** The Mysida maxilla consists of the common sympod (praecoxa, coxa, basis) and developed endopods and exopods (Fig 3C). Leaf-like in appearance, the maxilla functions as both a feeding-related filter and to produce a current for respiration. The praecoxa is small and does not bear a lobe. The coxa is produced inwardly to form a pronounced foliaceous lobe, set with a varying degree of setae and/or spines and fringed with a closely set row of plumose setae. The basis is produced into a chitinized lobe that is, with the exception of the genus *Petalophthalmus*, incised medially so that it seems to consist of two lobes, both bearing several rows of strong setae at their apex, seen as spinose setae in the Stygiomysida family Stygiomysidae (Fig 3E). Concerned with maintenance of the respiratory current, the maxilla exopod is always well-developed in the Mysida. The exopod is seen as a large plate attached to the outer side of the basis, with the outer margin markedly convex and fringed with closely set setae. The endopod is two-segmented and set on the distal portion of the basis and the distal segment is usually fringed with setae along its entire outer margin.

### Thorax

**Fig 4:**
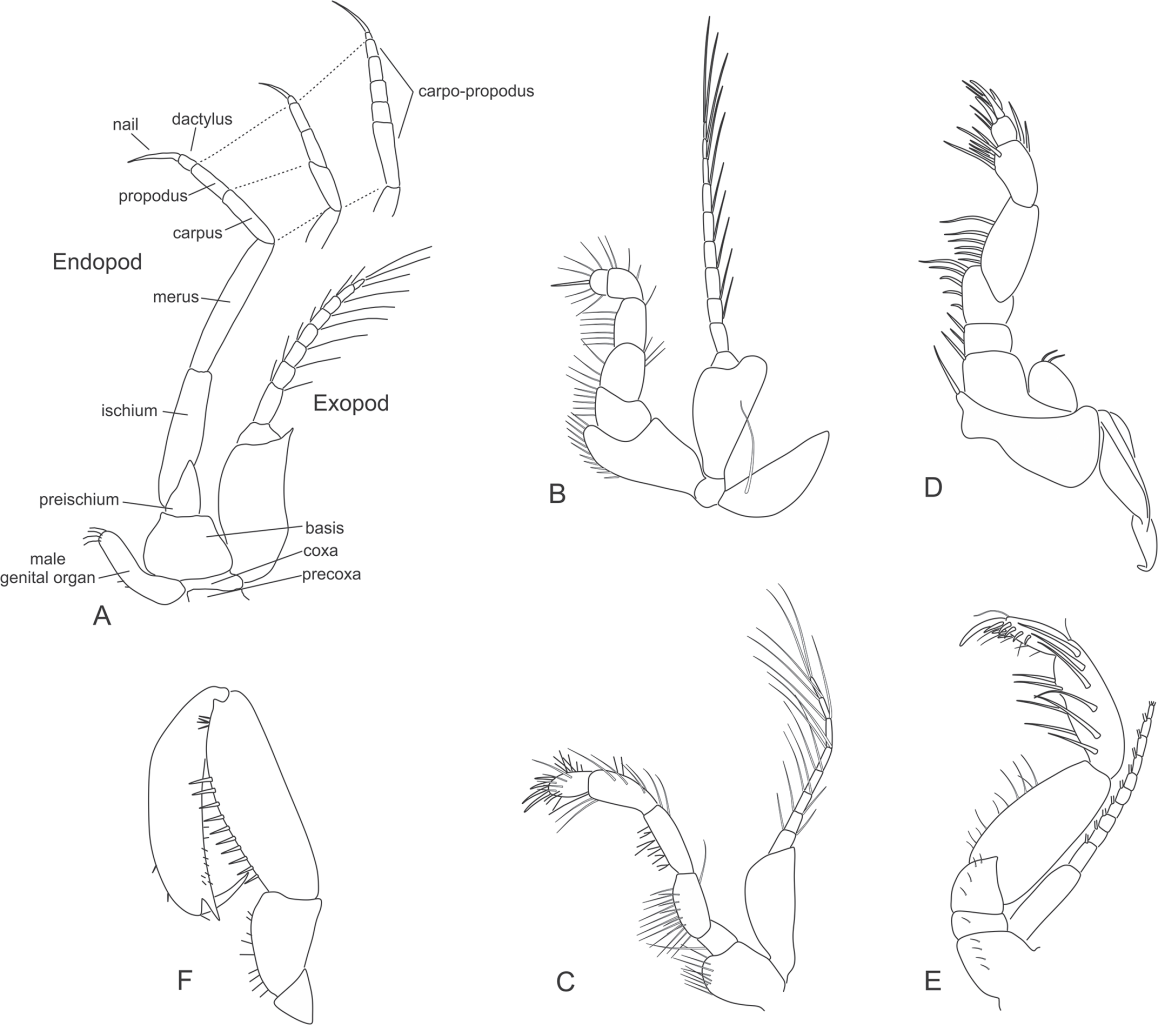
Mysida and Stygiomysida thoracopods. (A) General morphology. (B) thoracopod 1 (maxilliped), *Leptomysis gracilis* (G.O. Sars, 1864). (C) thoracopod 2 (maxilliped), *Mysis relicta* Lóven, 1862. (D) thoracopod 1 (maxilliped), *Stygiomysis holthuisi* (Gordon, 1958). (E) thoracopod 2 (maxilliped), *Stygiomysis holthuisi*. (F) thoracopod 3 (gnathopod); *Heteromysis microps* (G.O. Sars, 1877).

**1** ^**st**^ **thoracopod** The first pair of thoracic limbs differ considerably from the remaining thoracic appendages, modified for feeding as short, strong maxillipeds (Fig 4B). The base of this appendage consists of a two-segmented sympod composed of the coxa and basis. Arising from the coxa is a large lamellar epipod that produces a respiratory current. The thoracopod basis supports a well-developed exopod, consisting of a large flattened proximal segment and a multi-segmented flagellum. In the family Petalophthalmidae the exopod is completely reduced, and in the Stygiomysida and the Erythropinae genus *Mysimenzies* the exopod of the first maxilliped is reduced to an unsegmented lamina (Fig 4D). The endopod is short and robust, always closely associated with the mouth parts, and is slightly modified in segmentation where the carpus and propodus are fused to form what can be referred to as the carpo-propodus (alternative interpretation in Wittmann et al. [13]). Endites are mostly found on the basis and often on the ischium and merus. Ischium and merus are fused to form a merischium in the Leptomysinae genus *Mysidopsis*.

**2** ^**nd**^ **thoracopod** Similar to the first maxilliped, the second thoracopods always take on the form of maxillipeds, but bear no epipods (Fig 4C). The endopod supports a fused carpo-propodus. In the Stygiomysida the second thoracopods are enlarged, and the dactylus and nail bend down to form a subchelate gnathopod (Fig 4E). With the exception of the Petalophthalmidae genus *Petalophthalmus*, the second thoracic limb bears a well-developed natatory exopod composed of a large proximal segment (less pronounced in Stygiomysida) followed by a more slender segmented flagellum.

**3** ^**rd**^ **-8** ^**th**^ **thoracopods** are as a rule biramous with well-developed endopods and exopods, and no epipods. The endopod comprises six segments above the sympod: pre-ischium, ischium, merus, carpus, propodus, and dactylus (Fig 4A). The joint between the merus and carpus is sometimes referred to as a "knee". Distal to the "knee" the carpus and propodus may fuse to form a single segment. The combined carpo-propodus can also be secondarily divided into four or more sub-segments. In other taxa a subdivision is made up of a separate carpus accompanied by a secondarily subdivided propodus. Thoracopods of this type are used for swimming and/or walking and are often termed pereiopods.

In the Stygiomysidae, the third and fourth endopods have been modified to gnathopods. In the tribe Heteromysini the third thoracopod's dactylus is small and a powerful backwardly directed nail make a strong subchela at its distal end, taking on the form of a gnathopod (Fig 4F). These gnathopods can be used for seizing prey and passing it on to the mouthparts. In Rhopalophthalminae the eighth endopods show marked sexual dimorphism and are reduced to unsegmented vestiges. In male Mysida the genital organ is seen as a papilla (or as small lobes in Rhopalophthalminae) arising from the coxa of the eighth thoracopod. The Mysida exopods take on the common natatory form with a large proximal segment (less pronounced in Stygiomysida) followed by a more slender segmented flagellum. Respiration is a function of the inner surface of the carapace and there are no branchiae bearing appendages in the Mysida.

**Fig 5:**
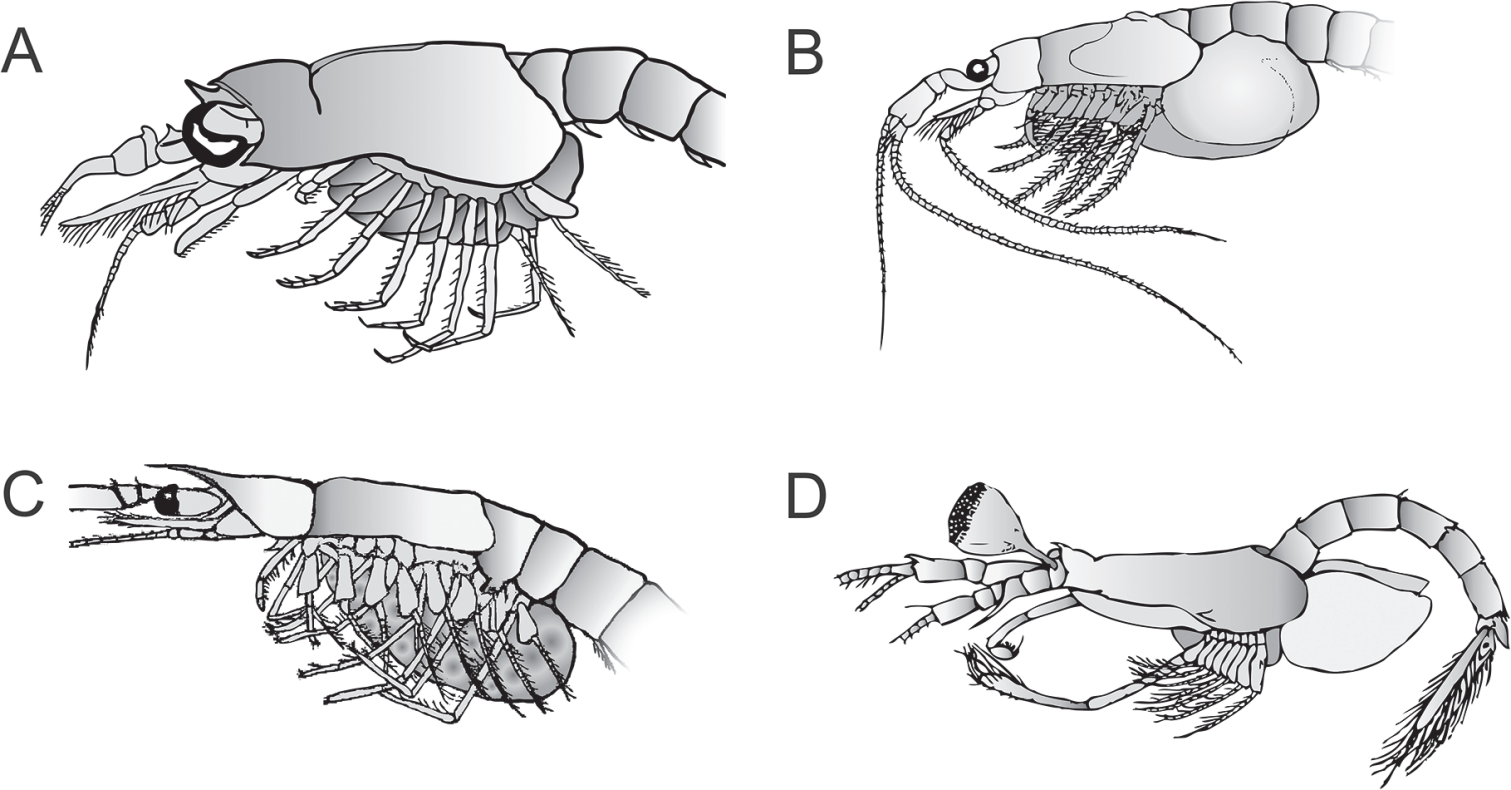
Mysida marsupium. (A) *Boreomysis tridens* G.O. Sars, 1870 (T&T, 1951.). (B) *Gastrosaccus sanctus* (van Beneden, 1861). (C) *Siriella armata* (Milne-Edwards, 1837). (D) *Arachnomysis leuckartii* Chun, 1887.

**Marsupium** The female marsupium is composed of oostegites arising from the bases and coxae of the thoracopods, forming a large brood pouch on the ventral side of the thorax (Fig 5). The oostegites are large, thin walled and concave plates, fringed with short, strong setae. In the Petalophthalmidae, Boreomysinae and in the Stygiomysida family Lepidomysidae the marsupium is composed of seven pairs of oostegites on the second to eighth thoracopods (Fig 5A). Within the family Mysidae a reduction of the anterior oostegites is seen, and the marsupium comprises two or three pairs of oostegites arising from the posterior appendages (Fig 5B). A unique marsupium composition is found in the Stygiomysidae where four pairs of oostegites arise from thoracopods three to six.

**Male genital organs** The mysidacean gonopores are located on the coxa of the eighth thoracopods. In the Stygiomysida male genitals consist of a closing apparatus formed by an anterior setose lobe and a posterior bare lobe that flank a genital orifice. In the Mysida, the gonopores are always well-developed. In the Petalophthalmidae genus *Hansenomysis* we find tubular penes with subterminal orifices that end in two apical lobes. This seems to be the general morphology of the male genital organs in the order Mysida. Variations between families and subfamilies are seen in penes size, number of apical lobes, and number and type of setae [14]. An exception to this general morphology is observed in the subfamily Rhopalophthalminae where non-elevated gonopores, comparable to the Stygiomysida, are armed with two small lobes on the inner distal margins of the enlarged coxa on the eighth thoracopods, coupled with reduced eight thoracic endopods. In the Mysidellinae the tubular penes are quite conspicuous in being long and slender extending anteriorly along the entire length of the thorax. The highest diversity of male genitals between genera is found in the subfamily Heteromysinae.

### Abdomen

In early larval stages, which live entirely in the maternal brood pouch, the abdomen of the Mysida is composed of seven segments. During development into young juveniles the sixth and seventh segments fuse to form what is seen as the sixth abdominal somite in the adults. In Mysida the abdominal somites do not support pleural plates, with the exception of females in the subfamily Gastrosaccinae where the pleura of the first abdominal somite are expanded to large plates supporting part of the marsupial pouch (Fig 5C). A similar expansion of the pleura is found in males of the subfamily Rhopalophthalminae.

**Fig 6:**
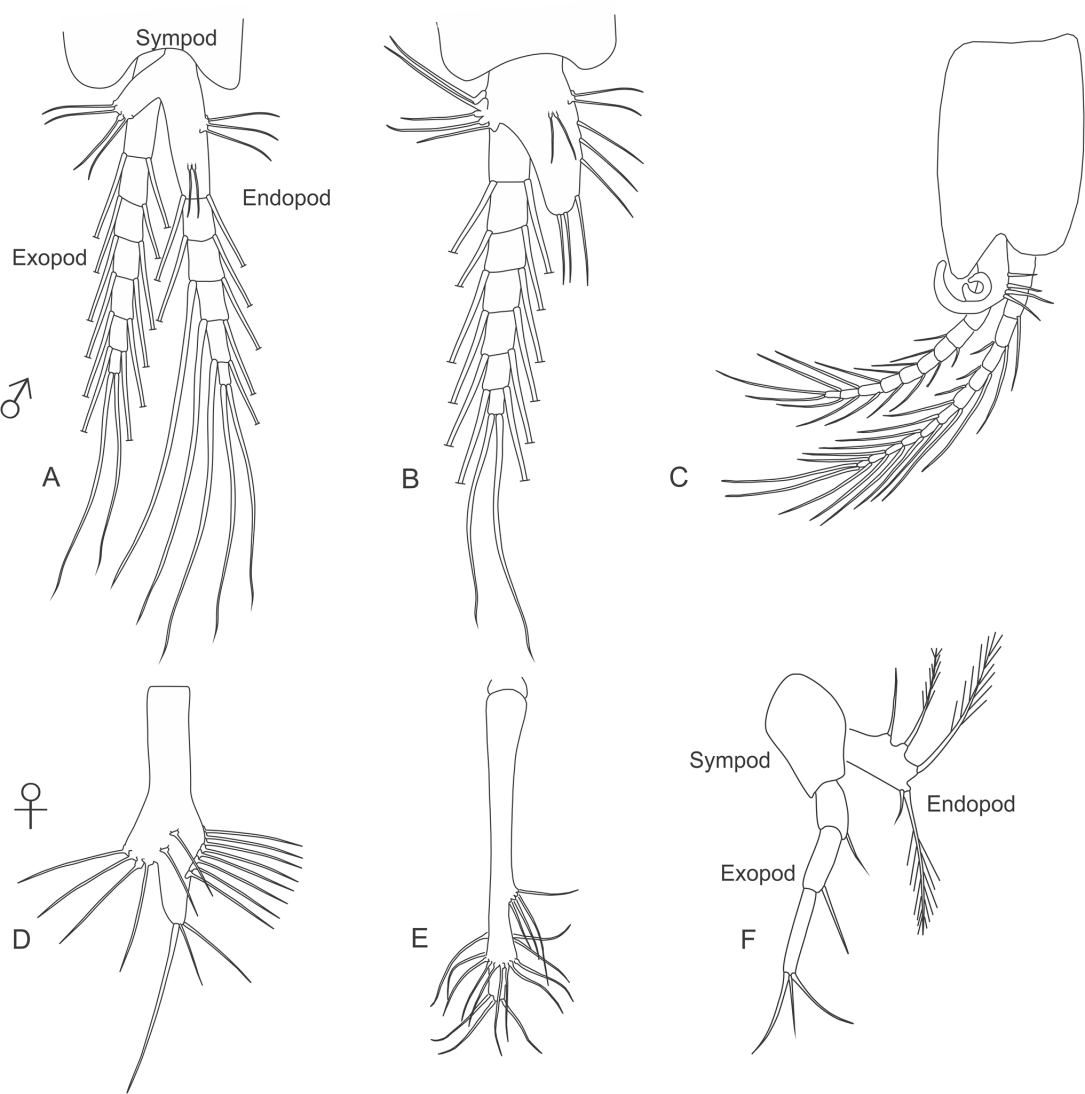
Mysida and Stygiomysida pleopods. (A) general morphology of male pleopod. (B) male pleopod with reduced endopod. (C) male pleopod 3, *Siriella armata* (Milne-Edwards, 1837). (D) general morphology of a reduced female pleopod. (E) female pleopod 1, *Hansenomysis fyllae* (Hansen, 1887). (F) female pleopod 2, *Stygiomysis holthuisi* (Gordon, 1958).

**Pleopods** A pair of pleopods is present on each of the first five abdominal somites. The basic pleopod is natatory in function, made up of a large flattened two-segmented sympod/protopod supporting two multi-articulated plumose rami, exopod and endopod. This basic construction is considered a plesiomorphic state for the Mysida (Fig 6A). As a rule the first pleopod endopod consists of a one-segmented plate and a normal exopod (Fig 6B). Unlike that found in the order Lophogastrida, the pleopods in Mysida species show marked sexual dimorphism. The female pleopods are usually reduced to simple, unjointed, setose plates (Fig 6D and 6E). Reduced male pleopods similar to unsegmented plates are also seen Heteromysinae, Mysidellinae, and Palaumysinae. In the Stygiomysida both male and female pleopods are reduced to comprise a sympod/protopod, a one-segmented endopod and three-segmented exopod (Fig 6F). The remaining Mysida taxa have biramous male pleopods that take on a variety of shapes and sizes; common for these pleopods are the pseudobranchiae at the base of each endopod. These pseudobranchiae take on the form of setose quadrangular and rounded plates, or spirally coiled branches (Fig 6C). For identification purposes general reductions in male exo- and endopods are very useful in determining subfamilies, and secondary modifications often seen in the third and fourth pleopods are quite valuable in identifying Mysida genera.

**Fig 7:**
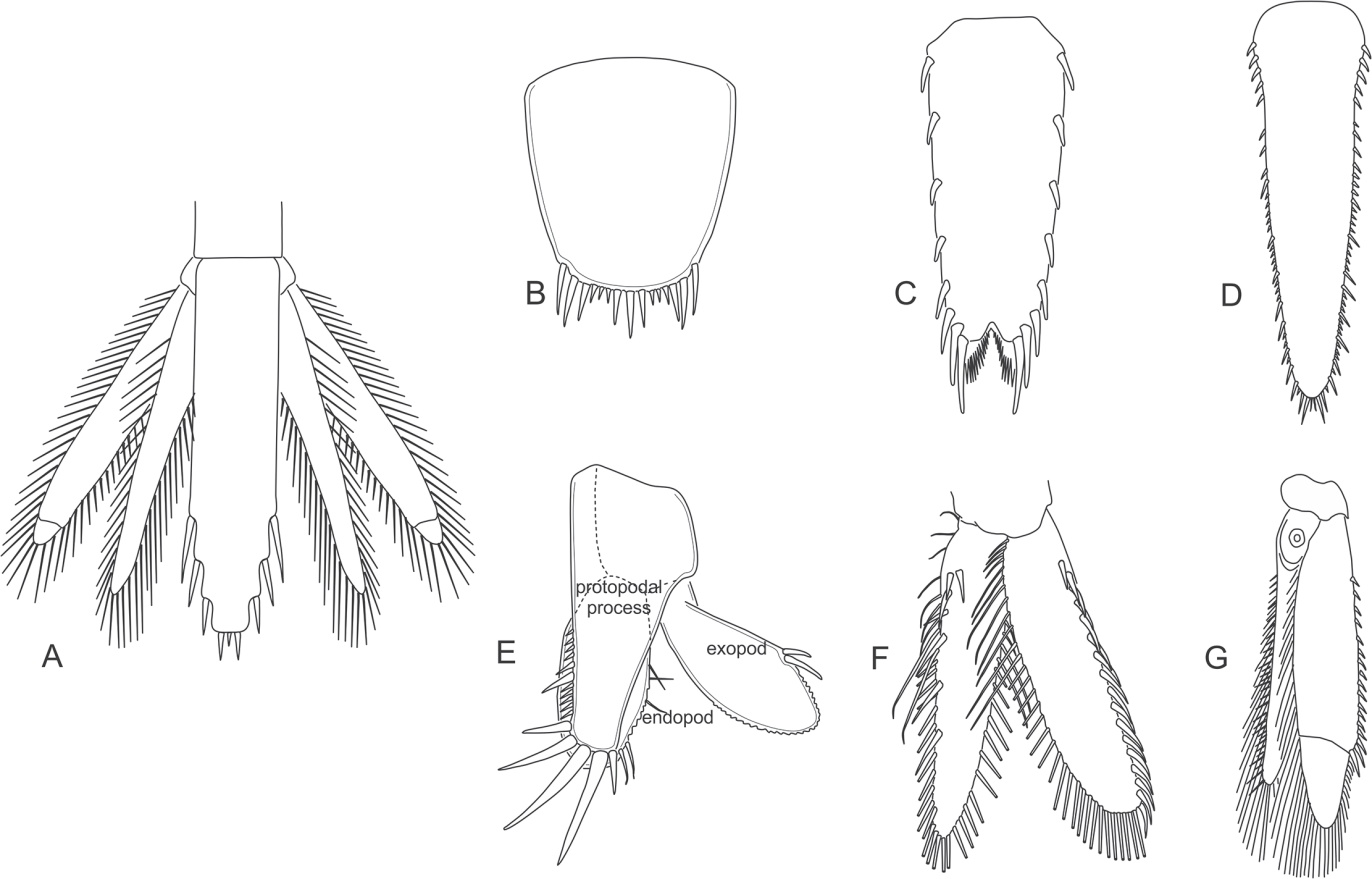
Mysida and Stygiomysida uropods and telson. (A) tail fan, *Hansenomysis fyllae* (Hansen, 1887). (B) telson, *Stygiomysis holthuisi* (Gordon, 1958). (C) telson, *Archaeomysis grebnitzkii* Czerniavsky, 1882. (D) telson, *Siriella norvegica* G.O. Sars, 1869. (E) uropods, *Stygiomysis holthuisi* (Gordon, 1958). (F) uropods, *Archaeomysis grebnitzkii*. (G) uropods, *Siriella norvegica*.

**Uropods** The last pair of abdominal appendages are made up of a two-segmented sympod that supports a large flattened exo- and endopod. The exo- and endopods are often divided by distal or proximal articulations (Fig 7A and 7G). The margins of both exo- and endopods are fringed with plumose setae and are often armed with a series of spiniform setae and/or spines, however are in some genera devoid of setae (Fig 7G).

**Statocyst** The proximal portions of the endopod of uropods are almost entirely occupied by a large balance organ, termed 'statocyst', appearing as a large, mostly clear vesicle, which arises as an invagination of the integument that contains a round, more or less flattened statolith (Fig 7G). These organs are exclusive of the family Mysidae (Mysida), not developed in the family Petalophthalmidae and order Stygiomysida (Fig 7A and 7E). The static organ serves as equilibrium organ for stabilization of body position and for directional swimming and provides information needed for stabilization of the visual field [15].

**Fig 8:**
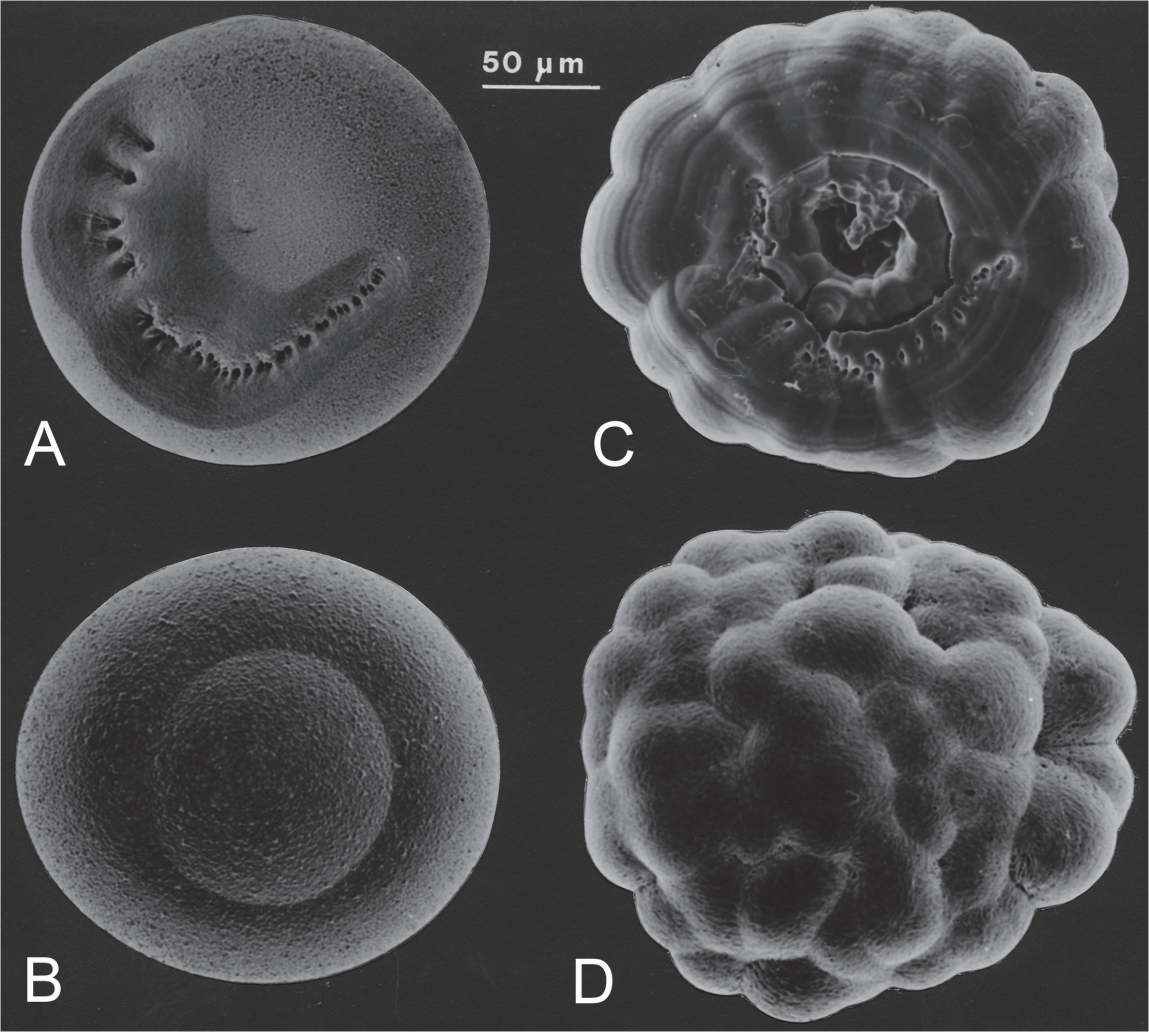
Mysidae statoliths. Mineralized with fluorite (A, B) or with vaterite (C, D). (A) *Schistomysis spiritus* (Norman, 1860), ventral view. (B) *Schistomysis spiritus*, dorsal. (C) *Schistomysis assimilis* (G.O. Sars, 1877), ventral. (D) *Schistomysis assimilis*, dorsal.

The statocyst cavity contains ambient water and a statolith that is renewed at each moult. Ventrally there is a sensory cushion bearing a radial series of sensory setae arranged in groups. These setae penetrate with their non-sensory apical portions into the large statolith. The statolith is commonly flattened and ellipsoidal, but may also be spherical, hemispherical, or moruloid [16]. The statolith is supported by the arch of the sensory setae from below, stimulating the setae by the effects of gravitation and inertia. Pores on the ventral face mark the openings of the mineral canals through which the setae penetrate into the statolith (Fig 8A and 8C). The sequence and grouping of the pores show characteristic patterns expressed as the 'statolith formula' [17], useful in identifying higher taxa of the Mysidae.

In about 86% of Mysidae species the statoliths are composed of CaF_2_ as the mineral fluorite (Fig 8A and 8B), mainly found in marine mysids. In about 9% of the Mysidae the statolith consists of CaCO_3,_ as the mineral vaterite (Fig 8C and 8D), most common in brackish and freshwater species [18]. The remaining 5% have non-mineralized (organic) statoliths. The organic statoliths are found in what is considered the less derived taxa, Rhopalophthalminae and Boreomysinae. Fluorite statoliths are found in all remaining subfamilies. Vaterite seems to be a derived character found in the subfamily Mysinae, where it is generally less frequent than fluorite, but often prevailing in taxa tied to specific biogeographical areas, particularly in the Ponto-Caspian [18, 19].

**Telson** Both the Stygiomysida and Mysida telson takes on a general form as a large flattened plate, where the anus always opens on the ventral side. Margins of the telson can be heavily serrated with spines, or fringed with strong setae. The apex may be either entire (Stygiomysida and Mysida) or cleft (Mysida) and in most cases armed with a varying degree of spines and/or setae (Fig 7A–7C).

## Fossil Record

After their first occurrence in the Devonian, the subclass Eumalacostraca had its main radiation in the Carboniferous, and it is during this period that at least two lineages of the “Mysidacea”, the Pygocephalomorpha and the Lophogastrida, first appeared in the fossil record. A large number of mysidacean fossils are preserved as moulds in Carboniferous to Permian sediments.

The majority of mysidacean fossils from the Palaeozoic are attributed to the extinct order Pygocephalomorpha. This is a species rich order with six families, comprising a total of at least 16 genera and 32 species. Due to seven pairs of oostegites in the female and the mostly large, carinate carapace in both sexes, the Pygocephalopmorpha are often classified as a suborder in the Lophogastrida [4, 20, 21]. Alternative hypotheses suggest a Eucarida placement [22, 23] or also simply belonging to the Eumalacostraca without assignment to a particular superorder [24].

The earliest fossil record from an extant order of mysidaceans is the Lophogastrida species *Peachocaris strongi* and *P*. *acanthouraea* from the Carboniferous of North America. Mainly based on the unmodified first thoracopods, similar in appearance to thoracopods two to eight, Schram [25] erected the Lophogastrida family Peachocarididae to accommodate these fossils. More reliable affinities with Lophogastrida are shown by the Triassic Eucopiidae species *Schimperella beneckei* and *S*. *kessleri* from deposits in France [26], and *S*. *acanthocercus* from China [21]. Proceeding into the Mid-Jurassic we find lophogastrids with strong similarity to extant genera, namely *Lophogaster voultensis* and *Eucopia praecursor* from deposits in France [23].

The earliest fossils attributed to the Mysida are the species *Elder unguiculata* and *Francocaris grimmi* from the Jurassic of Bavaria, Germany. However, Schram [25] doubted these records, as they are too poorly known. As in the Lophogastrida, there are fossil Mysida with amazing similarity to extant forms, namely *Siriella antiqua* and *S*. *carinata* from Mid-Jurassic deposits of France [23], and *Mysidopsis oligocenica* from the Oligocene of Italy [27].

The soft-bodied Mysida are generally poorly predisposed for fossilization. However, as described in the previous section, most species of the family Mysidae bear mineralized, endogenous statoliths as main components in their statocysts. Fossil statoliths were first recognized by Voicu [17, 28] in Miocene deposits of the brackish Paratethys, once extending from the Vienna basin to Lake Aral. These microfossils resemble the calcareous statoliths found in extant *Paramysis*, but are composed of the stable mineral calcite, most likely derived from the metastable vaterite through spontaneous phase transformation during fossilization [29]. To date, statoliths from three species of Miocene mysids are described. First attributed to the extant genus *Paramysis* [28], these statoliths are now thought to belong to the fossil genus *Sarmysis*.

## Taxonomy

### Establishing the Schizopoda

The Mysidae were first established by Haworth [30] to contain the genus *Mysis*. This genus had been instituted earlier by Latreille [31] for reception of species described by Fabricius [32]: the mysid *Cancer oculatus*, the leptostracan *Cancer bipes*, and an euphausiid species of obscure identity, *Cancer pedatus*. Additional species included *Praunus integer* (this was the original generic assignment for *Neomysis integer)*, and also the first published mysid, namely *Cancer flexuosus* by Müller [3] (now *Praunus flexuosus*). The latter two species had been reconsidered and placed in the genus *Mysis* by Leach [33], referring to species he just one year earlier had used to introduce the genus *Praunus* [34].

With support from Lamarck [35], a close relationship between *Nebalia* and *Mysis* was first suggested by Latreille [36] upon definition of the historical group Schizopoda for the reception of mysidacean and leptostracan genera. The Schizopoda were defined by a well-developed carapace, and biramous thoracopods bearing distinct endo- and exopods. Years later, Haworth [30] considered his group Mysidae equal in rank to Nebaliadae (now Nebaliidae), and placed both taxa as separate groups in the taxon Fissipedes (which has the same etymological meaning as the term Schizopoda). Based on uropod and telson morphology, Latreille [37] also recognized mysids and leptostracans as separate groups within the Schizopoda, and split the taxa accordingly.

For almost a century the Mysida were retained within the Schizopoda, and with reference to the classification of Anomobranchiata presented by Dana [38], where the Nebaliidae were excluded (Table 1), taxonomic controversy mainly concerned relationships between the Squilloidea and the remaining Schizopoda taxa.

**Table 1:** Classification according to Dana (1852) [38].

Order Anomobranchiata *sensu* Dana, 1852 (= Stomatopodae Milne Edwards, 1837)
Tribe (= suborder) Squilloidea
Tribe (= suborder) Mysidea
Family Euphausidae (*Thysanopoda*, *Euphausia*, *Cyrtopia*)
Family Mysidae
Subfamily Cynthinae (*Cynthia*)
Subfamily Mysinae (*Mysis*, *Macromysis*, *Promysis*, *Siriella*, *Loxopis*)
Subfamily Sceletinae (*Sceletina*, *Rachitia*, *Myto*)
Family Luciferidae
Tribe (= suborder) Amphionidea (*Amphionus*?)

During the mid-18^th^ century a multitude of new Mysida species were described. Noteworthy, the early works of J.V. Thompson [39, 40], who introduced the vernacular name "opossum shrimp" and established the mysid genus *Cynthia*, a junior homonym of the nymphalid butterfly taxon *Cynthia* Fabricius, 1807, and, therefore, later replaced by its junior synonym *Siriella* (Dana, 1850). Most important are the contributions from G.O. Sars’ species descriptions from Norwegian waters [41–43] and the Mediterranean Sea [44]. Pertaining to higher classification, G.O. Sars followed up on his father’s discovery of *Lophogaster typicus* M. Sars, 1857, by erecting the family Lophogastridae, which he placed within the Schizopoda, at equal rank with the Mysidae and Euphausiidae. G.O. Sars was reluctant to use Dana’s [38] classification and argued that the Schizopoda should be considered a suborder of the Decapoda, equally ranked with the suborders Brachyura, Anomura, and Macrura. He also drew attention to the apparent similarities of the marsupial plates in Mysidae and Lophogastridae with those found in Isopoda.

Reflecting new discoveries, the increased diversity of Mysida species lead to a first attempt on a comprehensive classification of the so far described Mysidae by Czerniavsky [45, 46] (Table 2). He followed the classification of Schizopoda by Willemoes-Suhm [47], and introduced the Schizopoda family Petalophthalmidae. The family Mysidae was divided into 6 subfamilies, where the Mysidellinae, Siriellinae, and Leptomysinae are still valid today and formally ascribed to Czerniavsky [45, 46]. Created by a tradition of overemphasizing minor infraspecific variations in separating species, many of Czerniavsky’s taxa were later classified as junior synonyms.

**Table 2:** Classification according to Czerniavsky (1882) [45].

Order Schizopoda *sensu* Czerniavsky, 1882
Family Nebalidae Willemoes-Suhm, 1875
Family Euphausidae Dana, 1852
Family Lophogastridae G.O. Sars, 1870
Family Petalophthalmidae Czerniavsky, 1882*
Family Mysidae Dana, 1852
Subfamily Mysidellinae Czerniavsky, 1882*
Subfamily Mysinae Czerniavsky, 1882
Subfamily Hemimysinae Czerniavsky, 1882
Subfamily Protomysidellinae Czerniavsky, 1882
Subfamily Siriellinae Czerniavsky, 1882*
Division (≈ tribe) Anchialidae Czerniavsky, 1882
Division (≈ tribe) Leptomysidae Czerniavsky, 1882*, **
Division (≈ tribe) Pontomysidae Czerniavsky, 1882
Subfamily Archaeomysinae Czerniavsky, 1882
Division (≈ tribe) Archaeomysidae Czerniavsky, 1882
Division (≈ tribe) Protomysidae Czerniavsky, 1882
Family Chalaraspidae Willemoes-Suhm, 1875

*Indicates those of Czerniavsky’s taxon names that are considered valid today.

**Currently accepted as the subfamily Leptomysinae Czerniavsky, 1882.

A few years later the controversy reached a breaking point. G.O. Sars [48] continued his arguments for the Schizopoda within the Decapoda, and at this point removed the Stomatopoda, which are now restricted to contain the Squilloidea, from the Schizopoda and Decapoda. Stebbing [49] followed up on Sars' hypothesis by defining the Malacostraca order Podophtalma to contain: Brachyura, Macrura, and Schizopoda, but disagreeing with Sars also included the Stomatopoda. An opposition to the Schizopoda concept had been supported already a few years earlier by Boas [50]. Based on homology of appendages within the Crustacea, Boas seriously questioned an affiliation between Euphausiacea and Mysidacea. In effect he split these taxa into two equally ranked orders within the Malacostraca, thereby rejecting the Schizopoda *sensu* G.O. Sars, 1870 [41], but he retained the Lophogastrida and Mysida as suborders within the Mysidacea and recognized the Eucopiidae as closely related to *Nebalia*. Hansen [51–53] took these ideas one step further in considering the Euphausiacea closely allied to the Decapoda and the Mysidacea to the Cumacea, Amphipoda, Isopoda, and Tanaidacea. Calman [20] respectively termed these taxa Eucarida and Peracarida, and with Calman's "On the classification of the Crustacea Malacostraca" the taxon Schizopoda was formally abandoned.

In the midst of discussions on higher taxonomy, other carcinologists were concentrating on the internal classification of the Mysidae. In his monograph on British Mysidae, Norman [54, 55] established the Gastrosaccinae, Heteromysinae, and must also be considered the one who defined, as we recognize them today, the taxa Siriellinae (under the junior synonym Cynthilinae) and Mysinae. Holt & Tattersall [56] erected the Boreomysinae. Tattersall [57] erected the subfamily Calyptomminae for the reception of *Calyptomma puritani*, which was abandoned two years later with the description of *Michthyops parva* (now *M*. *parvus*; senior synonym of *Pseudomma parvum*) [58], where both genera were placed in the Erythropinae.

After a gradual acceptance of a peracarid Mysidacea, Hansen [59] published a complete classification of the Mysidacea in his brilliant monograph "Schizopoda of the Siboga Expedition". Not only did Hansen introduce the Rhopalophthalminae, he also split the Leptomysini by erecting the Erythropini (Table 3). In retrospect Hansen's work, comparable to that of G.O. Sars, must be considered the most comprehensive morphological study on Mysidacea to date, and his classification has been widely used in crustacean taxonomy up to just recently (Table 3).

**Table 3:** Classification of the Mysidacea *sensu* Hansen, 1910, with additions by later authors.

Order Mysidacea Boas, 1883
Suborder Lophogastrida G.O. Sars, 1870
Family †Peachocarididae Schram, 1986*
Family Lophogastridae G.O. Sars, 1870
Family Gnathophausiidae Udrescu, 1984*
Family Eucopiidae G.O. Sars, 1885
Suborder Mysida Boas, 1883
Family Lepidomysidae Clarke, 1961*
Family Stygiomysidae Caroli, 1937*
Family Petalophthalmidae Czerniavsky, 1882
Family Mysidae Haworth, 1825
Subfamily Boreomysinae Holt & Tattersall, 1905
Subfamily Thalassomysinae Nouvel, 1942*
Subfamily Siriellinae Czerniavsky, 1882
Tribe Siriellini Czerniavsky, 1882
Tribe Metasiriellini Murano, 1986*
Subfamily Gastrosaccinae Norman, 1892
Subfamily Rhopalophthalminae Hansen, 1910
Subfamily Mysinae Haworth, 1825
Tribe Aberomysini Băcescu & Illife, 1986*
Tribe Calyptommini Tattersall, 1909**
Tribe Erythropini Hansen, 1910
Tribe Leptomysini Czerniavsky, 1882
Tribe Mysini Haworth, 1825
Tribe Heteromysini Norman, 1892
Tribe Mancomysini Băcescu & Iliffe, 1986*, ***
Subfamily Mysidellinae Czerniavsky, 1882

Authorships revised according to Art. 50.3.1 (ICZN 1999) [60].

*Taxa added after 1910.

**Hansen did not consider placement of Calyptommini. This taxon was reinstituted by Nouvel *et al*. [12].

***Not based on any generic name, therefore representing a nomen nudum.

### Expanding diversity and questioning the tribes

**Fig 9:**
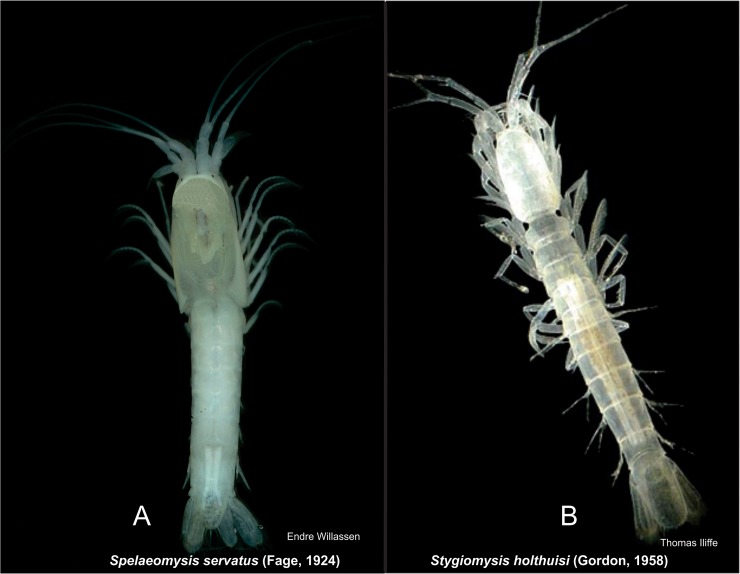
Species of Lepidomysidae (A) and Stygiomysida (B).

More recent taxonomic revisions within the Mysida have involved the additions and revisions of tribes, subfamilies, and suborders, with much of the new taxonomic structure involving the discovery of groundwater species (Fig 9). Upon the discovery of *Spelaeomysis servatus* (Fage, 1925) [61], the classification of groundwater species remained uncertain. Further discoveries of groundwater species led to the establishment of families Stygiomysidae (Caroli, 1937) [62] and Lepidomysidae (Clarke, 1961) [63], although the systematic position of these two families was not clear. Gordon [64] suggested a close affinity between these organisms, but recognized several ambiguities in external morphology and was therefore reluctant to assign these taxa to either Lophogastrida or Mysida. Characters linking the Stygiomysida to the Lophogastrida include foregut characters [65], while reduced female pleopods link them to the Mysida. Due to this unique suite of characters, Tchindonova [66] erected a new suborder, Stygiomysida, for these two families and revised the entire order Mysidacea accordingly. She also elevated the family Petalophthalmidae and subfamily Boreomysinae to the levels of suborder and family, respectively. More recent discoveries of groundwater species [67, 68] led to the establishment of the tribes Aberomysini and Mancomysini (now as Palaumysinae), which were considered to be members of the subfamily Mysinae. Molecular studies have supported the monophyly of the tribe Mancomysini (i.e. Palaumysinae), defined morphologically by uniramous male pleopods and a reduced antennal scale [6]. In contrast, members of the Aberomysini have not yet been included in molecular studies and morphological studies have found no support for the tribe, indicating the placement of *Aberomysis muranoi* within the Erythropini [12]. Finally, in the recently proposed classification scheme of the Mysida, the Mysinae tribes (e.g. Aberomysini, Erythropini, Leptomysini, Mysini, Heteromysini, Mancomysini) have been elevated to subfamily level while the genera of the tribe Calyptommini have been moved to the Erythropinae [6].

Although there have been additions and revisions, the taxonomy of the higher Mysida have remained relatively stable. In contrast, the monophyly of the ‘Mysidacea’, and the placement of the major mysidacean lineages, have remained controversial. Historically, conflicting ideas on Malacostraca phylogeny have often involved issues concerning the monophyly of the Mysidacea and Peracarida. Part of this debate stems from the concept of the “*caridoid facies*”. After the caridoid concept [2, 20], the taxonomic schemes within the Eumalacostraca have remained similar until the 1980s, when the monophyly of the Peracarida became an issue of debate. Central to these discussions was the placement of the ‘Mysidacea’. Within the mysidacean groups, the defining caridoid characters, coupled with the presence of oostegites, are plesiomorphic character states that do not support a monophyletic Lophogastrida-Mysida lineage. It was proposed that the Lophogastrida and Mysida were likely derived from separate peracarid ancestors [69], leading to proposals of splitting the Mysidacea into separate orders [24, 70]. Watling [71] argued that the ‘Mysidacea’ were paraphyletic, and that without the mysidaceans the Peracarida remained monophyletic. Further morphological studies showed that while the relationships among the mysidacean lineages are uncertain, the ‘Mysidacea’ are definitely within the Peracarida [72, 73]. The most current classification schemes reflect these studies, with the Lophogastrida and Mysida raised from suborder to order rank within the Peracarida [71, 74–76]. Recent phylogenetic studies of the Mysidacea, and their placement within the Malacostraca, have also begun to investigate these issues (see next section).

### “Mysidacea” and peracaridan affinity

Phylogenetic studies of the Mysidacea and their placement within the Crustacea have continued the debate over systematics and classification, with much of the disagreement occurring between morphological and molecular phylogenies. For the past decade the most widely accepted taxonomy of mysidaceans divided them into two major lineages, the Lophogastrida and the Mysida, subdivided into three and four extant families, respectively (Table 3). The Mysidae, within the suborder Mysida, contained the largest diversity of species, and were further divided into seven subfamilies and nine tribes (Table 3).

In the broader context of mysidacean placement within the Crustacea, morphological inference by Richter & Scholtz [73] supports a monophyletic Mysidacea within the Peracarida. According to De Jong-Moreau & Casanova [65], foregut morphology additionally supports the unity of the Mysidacea and demonstrates a gradual morphological transition from the Lophogastrida to Mysida through Petalophthalmidae and a separate lineage of Stygiomysidae from Lophogastrida ancestors.

In contrast, many of the molecular phylogenetic studies of the ‘Mysidacea’ have found significant incongruence between phylogenetic and taxonomic structure [77, 78]. Molecular studies of the ‘Mysidacea’ agree that the Lophogastrida and Mysida are not monophyletic, suggesting that the Mysida do not pertain to the Peracarida, and that the Stygiomysida (Lepidomysidae and Stygiomysidae) are not within the Mysida [6, 77, 79]. These studies particularly demonstrate the polyphyly of the ‘Mysidacea’ as currently delineated, and also illustrate the need for taxonomic revision within the Mysida.

### Phylogeny and current classification of the Mysida

**Fig 10:**
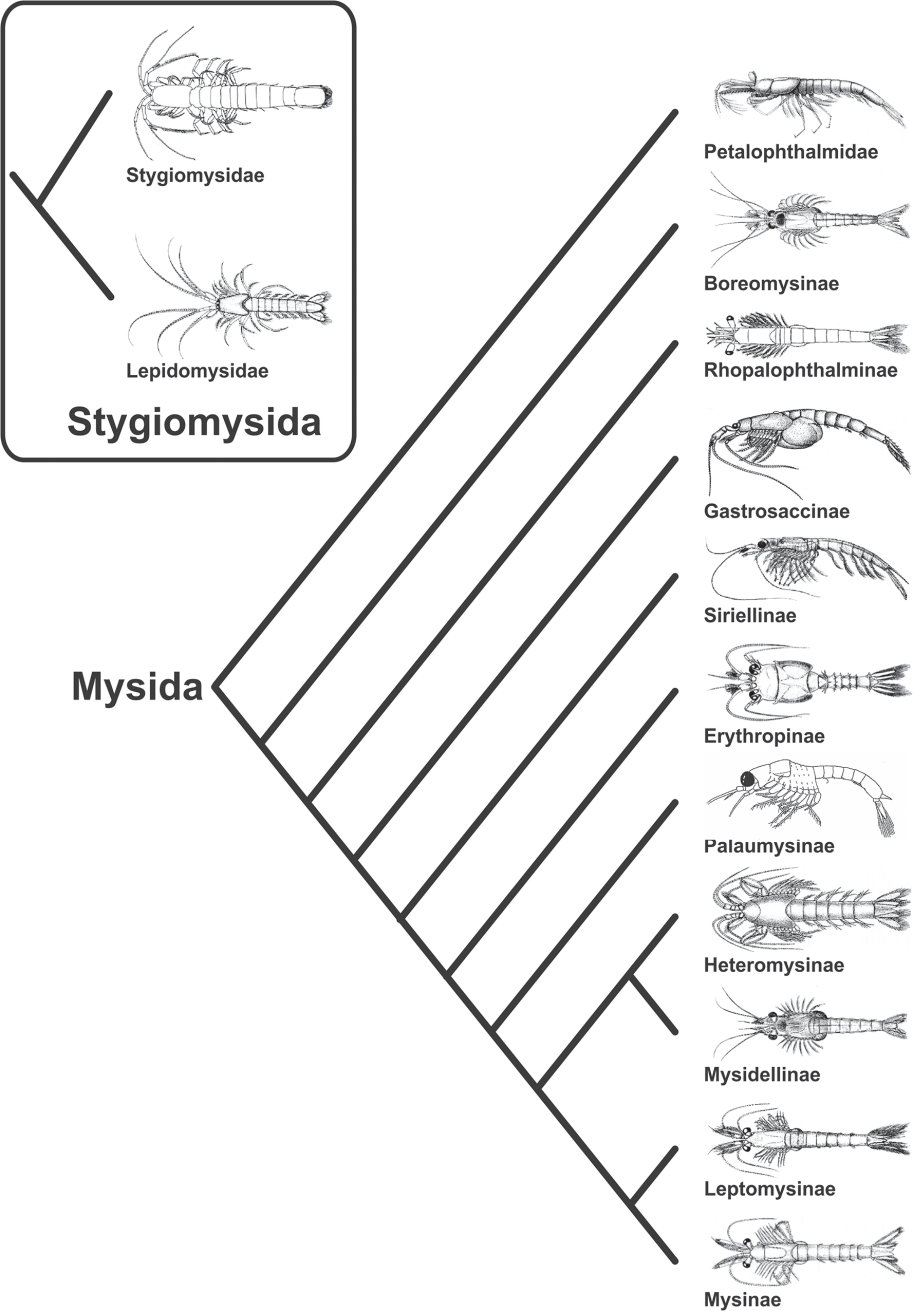
Proposed phylogeny of the Stygiomysida and Mysida (Meland & Willassen [6]).

Supporting the hypotheses first proposed by Gordon [64], molecular based analyses on the Malacostraca reveal the Lepidomysidae as closely related to the Stygiomysidae, tightly nested within the Peracarida, but without affiliation to the remaining Mysida taxa. With additional support in morphology [6] the Stygiomysida and Mysida (Fig 10) are acknowledged as separate orders (Table 4). Additional subdivisions of Mysidae subfamilies in Table 4 are available down to tribus level in Wittmann et al. [13].

**Table 4:** Classification of the Stygiomysida and Mysida according to Meland & Willassen [6]; with modifications by Wittmann [8] and Wittmann et al. [13] (tribes not shown).

Order Stygiomysida Caroli, 1937
Family Lepidomysidae Clarke, 1961
Family Stygiomysidae Caroli, 1937
Order Mysida Boas, 1883
Family Petalophthalmidae Czerniavsky, 1882
Subfamily Petalophthalminae Czerniavsky, 1882
Subfamily Hansenomysinae Wittmann, Ariani & Lagardère, 2014
Family Mysidae Haworth, 1825
Subfamily Boreomysinae Holt & Tattersall, 1905
Subfamily Rhopalophthalminae Hansen, 1910
Subfamily Siriellinae Czerniavsky, 1882
Subfamily Gastrosaccinae Norman, 1892
Subfamily Erythropinae Hansen, 1910*
Subfamily Leptomysinae Czerniavsky, 1882
Subfamily Mysinae Haworth, 1825
Subfamily Palaumysinae Wittmann, 2013**
Subfamily Heteromysinae Norman, 1892
Subfamily Mysidellinae Czerniavsky, 1882

*The monotypic taxa Aberomysinae Băcescu & Iliffe, 1986, Thalassomysinae Nouvel, 1942, and Mysimenziesinae Tchindonova, 1981, are not acknowledged at subfamily level and based on morphological evidence placed within the Erythropinae.

**Replacement name for the nomen nudum Mancomysinae Băcescu & Iliffe, 1986.

#### Stygiomysida

The Stygiomysida are a small group of subterranean groundwater mysidaceans comprising two monogeneric families, Lepidomysidae (9 *Spelaeomysis* species) and Stygiomysidae (7 *Stygiomysis* species) (Fig 9).

In the Stygiomysidae, the carapace is completely fused to abdominal tergites 1 to 4 and the four free thoracic somites pass imperceptibly into the abdomen (Fig 9B). This vermiform body-shape is quite common in stygobitic crustaceans, where the Stygiomysidae contain endemic species confined to anchihaline caves in the Caribbean Sea, Mexico, and Italy.

The Lepidomysidae are more mysid-like in appearance, with a clearly defined thorax partially enclosed by a posteriorly extending carapace and a clearly defined thorax and abdomen (Fig 9A). In addition to some species sharing a subterranean distribution with the Stygiomysidae, other species of Lepidomysidae are less confined stygophiles, and have been found in interstitial coastal belts, land crab burrows, and prawn culture fields.

Defining characters for the order Stygiomysida are biramous male and female pleopods, transverse lamellae from the posterior sternal margins of the abdomen, and elongated uropod protopodites. The presence of oostegites and absence of podobranchiae, coupled with non-statocyst bearing uropods have earlier been used to classify the Stygiomysida as primitive Mysida families, comparable to Petalophthalmidae [64]. Equally suggestive characters, but for a non-Mysida affiliation is seen in Stygiomysida displaying archaic foregut characters comparable to those found in the Lophogastrida [65]. Turning to molecular based phylogenies, DNA sequence data of ribosomal genes clearly demonstrate a sister group relationship between Stygiomysidae and Lepidomysidae as closely related to Lophogastrida and Hirsutidae (Mictacea) within the Peracarida, far removed from the Mysida [6].

Future research concerned with the evolutionary history of Peracarida taxa will certainly help to resolve the phylogenetic classification of Stygiomysida. Until then, based on combined morphological and molecular evidence the Mysida and Stygiomysida are to be treated as separate orders.

#### Mysida

The Mysida comprise two families, the Petalophthalmidae, which are mysids that do not have a uropodal statocyst; and the family Mysidae, which are the commonly known statocyst bearing mysids.

The Petalophthalmidae (6 genera, 39 species) are confined to deep-sea habitats and have adapted a raptorial feeding habit. In this regard, modification in mouthpart morphology is seen in the loss of a filter plate on the maxilla, development of meral lobes on the endopods of the second thoracic limbs, and also reduction of the first and second (in *Petalophthalmus*) thoracopod exopods.

An ancestral placement of the Boreomysinae within the statocyst bearing family Mysidae is supported by the presence of a female marsupium comprising seven pairs of oostegites, and that all male pleopods are biramous. The remaining Mysidae taxa have two or three pairs of oostegites and a varying degree of reductions in the male pleopods. Strong support for an ancestral Boreomysinae is also seen in the non-mineralic (organic) composition of the uropodal statoliths, a trait also found in the basal taxa Rhopalophthalminae. In the remaining Mysidae taxa the statoliths are mineralized with either fluorite or calcium carbonate [18].

An additional shared character state in the basal taxa Petalophthalmidae, Boreomysinae, Rhopalophthalminae, and also in the more derived Siriellinae, is the presence of a suture in the exopod of uropod. With reference to molecular phylogeny (Fig 10), the divided exopod gains support as an ancestral state in Mysida evolution.

The Gastrosaccinae are a mysid group predominately comprising species that have specialized in burrowing immediately under the sediment surface. A habitat-related autapomorphy and defining character for the Gastrosaccinae is seen in the female’s first abdominal somites having the pleural plates developed into a pair of lateral lamellae that take part in the formation of a strong brood pouch. The phylogenetic placement of Gastrosaccinae, falling basal to the Siriellinae is largely based on the apomorphic presence of not more than three pairs of oostegites combined with strong molecular support.

The Siriellinae are considered derived Mysidae and form a strong sister-group relationship with a large clade comprising the previously defined subfamily Mysinae tribes *sensu* Hansen, 1910 [59], and subfamily Mysidellinae. An autapomorphic character defining the Siriellinae is the presence of a spirally coiled pseudobranchia on the male pleopods in most species. However, besides a tendency towards modifications and reductions of male pleopods, strong morphological evidence supporting a Siriellinae relationship to higher Mysida remains to be found.

The aforementioned lack of morphological synapomorphies defining the Siriellinae placement within and relationships to other Mysidae clades is a conundrum shared with few exceptions by most higher Mysidae taxa (Siriellinae, Erythropinae, Leptomysinae, Mysinae, Heteromysinae, Mysidellinae, and Palaumysinae). To date no single apomorphic character has been found to define the subfamily Mysinae *sensu* Hansen, 1910 [59], but have instead been taxa loosely defined by retention of selected plesiomorphies. In effect, placements of certain genera, especially between tribes Mysini and Leptomysini (Table 3), have been riddled with revisions. In the current phylogeny-based classification, the tribes of Hansen [59] are elevated to subfamily level (Table 4), where support for monophyletic subfamilies is founded through morphological inference and on DNA-sequence based clades [6]. Defining morphology comprises a unique combination of shared morphological, albeit not necessarily apomorphic, character states, including antennal plate structure, pereiopod segmentation, pleopod reductions, and telson shape and armature.

Despite the unique “mantis-like” appearance of Heteromysinae species, owing to the third thoracopod where the nail bends over the dactylus forming a strong prehensile claw, a close relationship to the less conspicuous Mysidellinae has been made evident in highly similar 18S rDNA. Their relationship is manifested in that both the male and female pleopods are reduced to rudimentary plates, and also that in both taxa the male genital organs are seen as anteriorly produced cylindrical tubes. The Mysidellinae in turn show a strongly asymmetric labrum representing a clear autapomorphy.

## Biogeography

**Fig 11:**
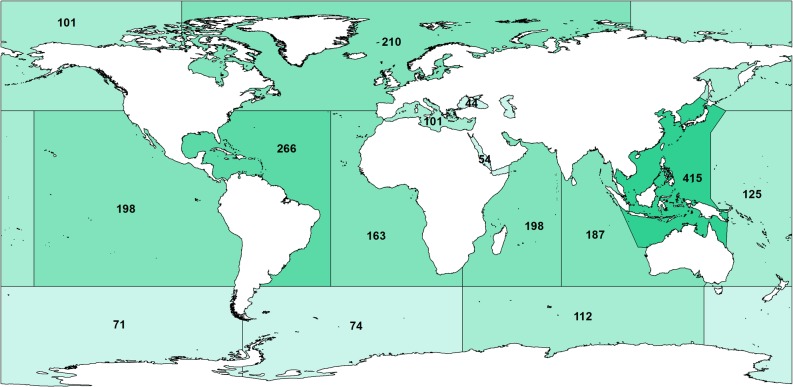
Number of known Mysida species recorded from the geographical regions proposed by Mauchline & Murano [91]

Among the three “Mysidacea” orders, the Lophogastrida are exclusively marine, the Mysida mostly so, and the Stygiomysida only marginally (Fig 11). The Stygiomysida most likely retreated into subterranean brackish and fresh-water habitats during the Tertiary, possibly earlier. Porter et al. [80] reviewed the invasion of certain Mysida and most Stygiomysida into fresh- and oligohaline waters (<3g/l) of the Palaearctic and Neotropical regions. Here, non-marine biogeographical groups are defined as subterranean Tethyan relicts, autochthonous Ponto-Caspian endemics, glacial relict species (*Mysis*), and euryhaline estuarine species. Regarding the Lophogastrida and the Mysida, biogeographical subdivisions and depth distribution have been studied by Petryashev [81–83] for the Arctic, North Atlantic, North Pacific and the (Sub)-Antarctic, and by Daneliya & Petryashev [84] for the Ponto-Caspian.

### Stygiomysida

This small order comprises mainly cavernicolous and/or phreaticolous species. The animals are found mostly in fresh- to brackish subterranean waters, however *Spelaeomysis cardisomae* lives in small pools at the bottom of crab burrows close to the margin of mangrove stands [85], and *S*. *cochinensis* occurs in prawn culture fields of India [86]. Most species are commonly confined to complete darkness, but some of the essentially photophobic species are on occasion found in weakly illuminated habitats. This is examplified by *S*. *bottazzii* from brackish groundwater in Apulia (SE-Italy), which approachs the margins of the photic zone mainly for feeding, and seek the deep, dark groundwater probably as shelter during the long incubation of young [29]. The Lepidomysidae comprises the genus *Spelaeomysis*, and shows a clear circumtropical distribution, but is missing in the central and western Pacific. The Stygiomysidae, with its only genus *Stygiomysis*, is restricted to Mediterranean and Caribbean waters [87]. The actual distribution of this family follows the path of the former Tethyan Sea [88], which extended during the Mesozoic from today's Caribbean eastwards to the Indian Ocean.

### Mysida

In general the Mysida are largely epi- to hyperbenthic organisms, *i*.*e*. living either on or hovering closely above the sea floor. There are a few holopelagic species of the family Mysidae, and some of these have a nearly circumtropical distribution (e.g. *Anchialina typica*, *Siriella thompsonii*). These species are essentially epipelagic, but the majority of pelagic species are more commonly represented by mesopelagic Erythropinae. Here we also find bathypelagic species that gradually move towards a more archibenthic mode of life. In these deep-water habitats we find some of the most species-rich cosmopolitan genera such as *Amblyops* and *Pseudomma* [89]. In terms of distribution the large subfamilies of the Mysidae are found around the world, nonetheless seem to be less diverse in polar regions. Species overlap between Arctic and Antarctic mysids is small, but there are examples of bipolar species that most probably transcend across the deep sea [82].

Species richness in the Indo-Pacific is generally higher when compared to the Atlantic [90]. With regard to benthopelagic and bottom dwelling mysids, the east and west coasts of both the Pacific and Atlantic oceans are greatly different regarding their warm-temperate and tropical faunas. And it is these warm coastal areas where we find the highest diversity, represented largely by the subfamily Mysinae. This pattern appears to be less strong in the Indian Ocean. Benthopelagic and benthic mysid species show endemism at all geographical scales, from specific sea basins (e.g. *Gastrosaccus mediterraneus* in the Mediterranean) down to single water bodies (e.g. the subterranean *Troglomysis vjetrenicensis* in a fresh-water cave at the east coast of the Adriatic Sea). Endemism is commonly less pronounced in temperate regions. Species diversity can be high, but we usually observe a broad distribution. In these areas species distribution is highly dependent on shared habitat preferences (depth, bottom type, salinity, temperature) and less on temporal and spatial distance.
